# Catalytic Oxidative Cleavage of C(OH)-C Bonds in Lignin Model Compounds to Carboxylic Acids by Fe(NO_3_)_3_.9H_2_O/NaI/DMSO

**DOI:** 10.3389/fchem.2022.933763

**Published:** 2022-07-01

**Authors:** Xuerong Wang, Huilin Sun, Caicui Li, Shuijiao Niu, Yu Gao, Ying Chen, Tianwei Xu, Jinhui Wang, Huanjun Xu

**Affiliations:** ^1^ Key Laboratory of Child Cognition & Behavior Development of Hainan Province, Qiongtai Normal University, Haikou, China; ^2^ School of Science, Qiongtai Normal University, Haikou, China; ^3^ Shandong Institute for Food and Drug Control, Ji’nan, China; ^4^ Department of Medicinal Chemistry and Natural Medicine Chemistry, College of Pharmacy, Harbin Medical University, Harbin, China

**Keywords:** catalytic oxidative cleavage, lignin model compounds, carboxylic acids, Fe(NO_3_)_3_.9H_2_O/NaI/DMSO, C(OH)-C bonds

## Abstract

The secondary C(OH)-C bonds are abundant in biomass such as lignin and cellulose. Thus, selective cleavage of the C(OH)-C bonds into value chemicals attracted much attention. Molecular iodine has received considerable attention as an inexpensive and readily available catalyst to yield the corresponding products in excellent yields with high selectivity, but it is highly corrosive and toxic, making its use somewhat unattractive. In this study, I_2_ was generated *in situ* from Fe(NO_3_)_3_.9H_2_O/NaI, which was further combined with Fe(NO_3_)_3_.9H_2_O to catalyze the oxidation process. In the reaction, the H_2_O molecule from the reaction and Fe(NO_3_)_3_.9H_2_O attacked the phenylglyoxal to form benzaldehyde, which was further oxidized to benzoic acid. Aryl primary and secondary benzylic alcohols from lignin were successfully transformed into aryl carboxylic acids by Fe(NO_3_)_3_.9H_2_O/NaI/DMSO. The catalytic system was green and efficient, avoiding the usage of toxic and corrosive molecular I_2_. From the experiments, it was clear that the yield of the product from the substrates with an electron-donating group was higher than that of electron-withdrawing substituted substrates, which was similar to the aryl secondary alcohols. Aryl alkyl ketones were also successfully conducted by the Fe(NO_3_)_3_.9H_2_O/NaI/DMSO catalytic system.

## Introduction

Lignin is the largest renewable source with a large number of aromatic units; thus, it could serve as a sustainable candidate feedstock for aromatic chemicals (Ohlrogge et al., 2009). Inspired by this, there are more efforts to selectively transform lignin into value-added chemicals, especially aromatic chemicals. In natural lignin, C(OH)-C bonds are widespread. Thus, it is an attractive strategy for selective cleavage and functionalization of C(OH)-C bonds for converting lignin into value-added chemical products, such as aryl carboxylic acid. Aryl carboxylic acid is very common in many structures of bioactive molecules and can be transformed to esters, amides, acid halides, and so on (Peng et al., 2016; Nicolaou et al., 2009; Liu et al., 2020). Although the C(OH)–C bonds in lignin have inherent kinetic inertness and thermodynamic stability, it is still very challenging for selective functionalization of these bonds.

In recent years, because molecular iodine possesses some advantages such as low in cost and easy to obtain, it has attracted considerable attention. More importantly, it is very efficient to make use as a powerful catalyst for various organic transformations with excellent yields and high selectivity under mild conditions, owing to its oxidation ability and Lewis acidity (Iida and Togo, 2006; Hazra et al., 2017). For example, Hazra’s group has reported a metal-free catalytic system with iodine/NaOH, which could transform alcohols and aldehydes into carboxylic acids with excellent yield (Hazra et al., 2017). It is clear that I_2_ is very useful in various types of organic reactions, but molecular iodine is highly corrosive and toxic, resulting in somewhat undesirable results in many reactions. Researchers conducted some excellent works to overcome the direct usage of I_2_. Bailey et al. have reported that CuSO_4_/NaI used in combination could be an *in situ* generation of I_2_ (Mohan et al., 2006). Subsequently, another convenient system to generate I_2_ *in situ* was reported by Tamin’s group, which consisted of Fe(NO_3_)_3_.9H_2_O/NaI, and the amount of I_2_ generated *in situ* was measured by UV/Vis spectrophotometry (Rostami et al., 2009). Also, this group has applied this protocol for trimethylsilylation and formylation of various alcohols catalyzed by I_2_ generated *in situ* from Fe(NO_3_)_3._9H_2_O/NaI (Amin et al., 2011).

Moreover, iron is low in cost and toxicity and has rapidly developed in various fields (Martin and Suarez, 2002; Plietker and Dieskau, 2009; Czaplik et al., 2010). Compared with other transition metals, most of the iron species show low toxicity (Perutz, 1979). The oral permitted daily exposure limit of elemental iron is high at 13,000 Ug/day; thus, the application of elemental iron in the pharmaceutical and food industries is attractive (Bauer and Knölker, 2015). In recent years, iron-catalyzed oxidation reactions to pharmaceutical molecules have attracted significant attention. Also, Li’ group reported a procedure of aerobic oxidative deoximation reactions that were catalyzed by iron (Li et al., 2018). In 2013, Ma’s group have reported Fe(NO_3_)_3._9H_2_O/TEMPO/MCl (M = Na or K) oxidized corresponding alcohols to carbonyl compounds using O_2_ or air as a terminal oxidant at room temperature (Jiang et al., 2016; Liu and Ma, 2013a; Liu and Ma, 2013b). Also, Xu and co-workers reported a strategy of I_2_/Fe(NO_3_)_3_.9H_2_O-catalyzed C–C bond cleavage of aryl alkyl ketones and secondary benzylic alcohols (Xu et al., 2018a). Notably, all these related research studies have confirmed that iron salts are efficient in catalytic oxidation reactions, but all the catalytic systems need other types of co-oxidants to promote catalytic activity.

Inspired by those finding, we wish to report a facile and efficient approach for the oxidation of secondary C(OH)-C bonds in lignin to aryl carboxylic acid by using cheap and nontoxic Fe(NO_3_)_3_ and NaI as a catalyst, in which I_2_ was generated *in situ* under the reaction, avoiding the usage of toxic and corrosive molecular I_2_. A variety of secondary and primary benzylic alcohols could be transformed into corresponding acids in moderate-to-excellent yields.

## Experimental Section

### Materials

All of the materials were purchased from Beijing Innochem Company and used as received.

### Characterization

Other than the 9H-fluoren-9-ol, mass spectra were obtained on an SCIEX X500R QTOF high-resolution mass spectrometry instrument with negative ion mode. The 0.1 acetic acid/5 mM amine acetate in CH_3_CN/H_2_O (20:80) was used as the eluent.

### General Procedures for Aerobic Oxidation

Typical procedure: the desired amount of secondary alcohol substrate (0.5 mmol), Fe(NO_3_)_3_.9H_2_O (0.15 mmol), NaI (0.075 mmol), and DMSO (2 ml) was added into a 25-ml reaction bottle. Then, the mixture was degassed three times with the oxygen balloon, and the reaction was held under 130°C for the desired time. After being acidified with 2 mol/L HCl (3 ml), the solution was extracted by ethyl acetate (5 ml) twice, and the organic phase was washed with saturated brine once and dried by Na_2_SO_4_. The combined organic phase was removed from the solvent by a rotary evaporator. The desired product was obtained through column chromatography using ethyl acetate/petroleum ether as an eluent.

## Results and Discussion

Initially, 1-phenyl-1-propanol was taken as the model substrate to optimize the reaction conditions (Table 1). We tried to optimize the reaction with a suitable amount and ratio of Fe(NO_3_)_3_.9H_2_O/NaI; when the catalytic system consisted of 20 mol% of Fe(NO_3_)_3_.9H_2_O and 10 mol% of NaI, the desired product could be successfully obtained with 67% yield in 18 h (Table 1, entry 1). When the loading of Fe(NO_3_)_3_.9H_2_O was further increased to 30 mol% and NaI was increased to 15 mol%, the yield was sharply increased to 83% (Table 1, entry 2). But, while maintaining the loading of Fe(NO_3_)_3_.9H_2_O at 30 mol%, the loading of NaI was further increased to 30 mol%, and then the benzoic acid was yielded at 72% (Table 1, entry 3). While other types of nitrate catalysts were used as catalyst, the yield of acid was lower than when the Fe(NO_3_)_3_.9H_2_O was used as the catalyst (Table 1, entries 4–5). We also test other types of salts as the co-catalyst, such as LiI, KI, NaBr, or NaCl, but all those salts cannot offer a satisfactory yield of acid (Table 1, entries 6–10).

**TABLE 1:** Optimization of reaction conditions^a^.

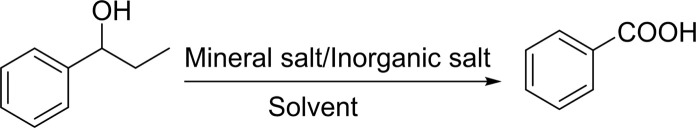				
**Entry**	**Mineral salt**	**Inorganic salt**	**Solvent**	**Yield (%)^b^**
1	Fe(NO_3_)_3_.9H_2_O	NaI	DMSO	67^c^
2	Fe(NO_3_)_3_.9H_2_O	NaI	DMSO	72^d^
3	Fe(NO_3_)_3_.9H_2_O	NaI	DMSO	83/0^e^
4	Cu(NO_3_)_2_	NaI	DMSO	17
5	Co(NO_3_)_2_	NaI	DMSO	11
6	Fe(NO_3_)_3_.9H_2_O	KI	DMSO	16
7	Fe(NO_3_)_3_.9H_2_O	LiI	DMSO	65
8	Fe(NO_3_)_3_.9H_2_O	NaCl	DMSO	56
10	Fe(NO_3_)_3_.9H_2_O	NaBr	DMSO	27
11	Fe(NO_3_)_3_.9H_2_O	NaI	DMF	12
12	Fe(NO_3_)_3_.9H_2_O	NaI	NMP	15
13	Fe(NO_3_)_3_.9H_2_O	NaI	Mesitylene	51
14	Fe(NO_3_)_3_.9H_2_O	NaI	DMSO	8^f^/81^g^
15	Fe(NO_3_)_3_.9H_2_O	NaI	DMSO	78^h^
16	Fe(NO_3_)_3_.9H_2_O	NaI	DMSO	82^i^
17	Fe(NO_3_)_3_.9H_2_O	—	DMSO	71^j^

a Condition: substrate (0.5 mmol), Fe(NO_3_)_3_.9H_2_O (0.15 mmol), NaI (0.075 mmol), DMSO (2 ml), and air balloon, 130°C, 18 h.

b Isolated yield.

c Substrate (0.5 mmol), Fe(NO_3_)_3_.9H_2_O (0.1 mmol), NaI (0.05 mmol).

d Substrate (0.5 mmol), Fe(NO_3_)_3_.9H_2_O (0.075 mmol), NaI (0.075 mmol).

e Under N_2_.

f 12 h.

g 24 h.

h 120°C.

i 140°C.

j Substrate (1 mmol), of I_2_ (0.1 mmol), Fe(NO_3_)_3_.9H_2_O (0.1 mmol), DMSO (2 ml), and O_2_ (0.1 Mpa) 130°C, 12 h.

A series of solvents including DMF, NMP, and mesitylene were screened, and it was clearly shown that DMSO was the best solvent among them (Table 1, entries 11–13). When the reaction was conducted in the absence of oxygen, the yield of benzoic acid was not detected, but benzaldehyde was formed with a 65% yield (Table 1, entry 3). Then, we proceeded with the reaction under different temperatures and times, and it was found that the yield of the product was lower under 120°C (Table 1, entry 15). The yield of the desired product was not improved when the reaction temperature was increased to 140°C or the reaction time was increased to 24 h (Table 1, entries 14 and 16). More interestingly, when the reaction time was decreased to 12 h, the yield of acid was just 8%, and most of them was benzaldehyde, indicating that benzaldehyde was transformed to the desired product faster.

Thus, it was obvious that NaI (15 mol%)/Fe(NO_3_)_3_.9H_2_O (30 mol%) as the catalyst and DMSO as solvent under O_2_ balloon at 130°C for 18 h could efficiently catalyze aerobic oxidation of alcohol. Subsequently, the standard reaction condition was chosen to explore the scope and generality of this protocol. The various aryl alkyl alcohols were further investigated, with the results shown in Table 2.

**TABLE 2:** Catalytic aerobic oxidation of secondary alcohols^a^.

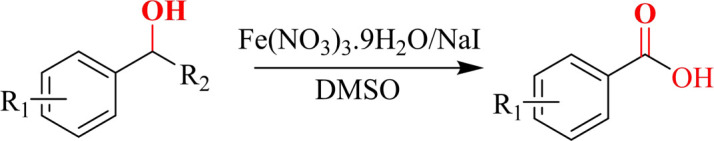		
**Entry**	**Substrate**	**Product/yield** ^b^
1	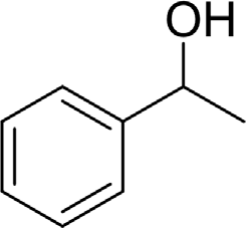	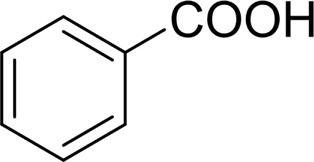 83
2	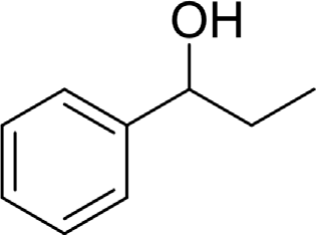	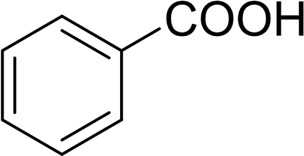 83
3	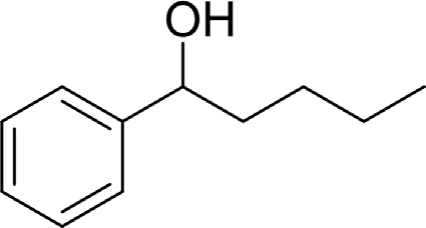	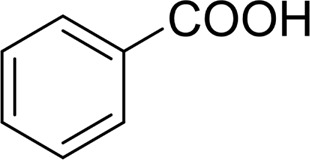 82
4	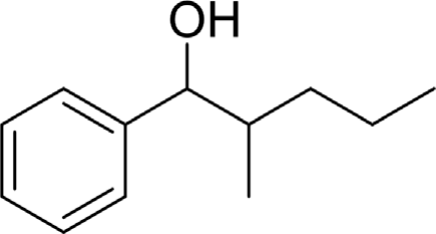	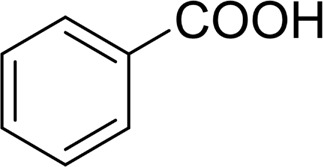 42
5	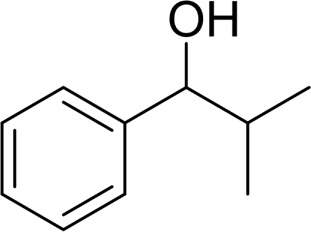	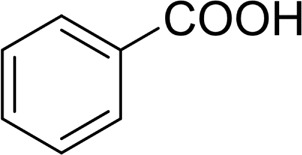 49
6	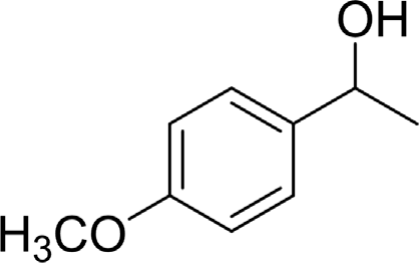	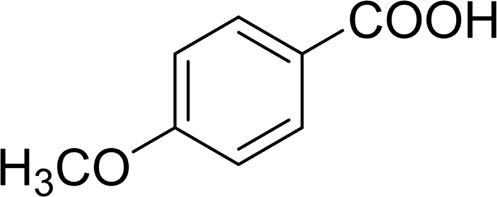 74
7	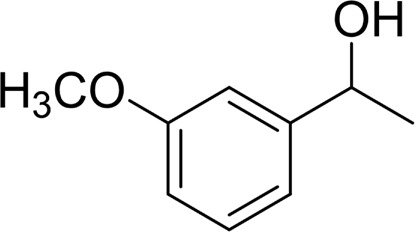	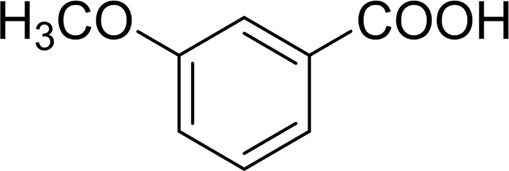 82
8	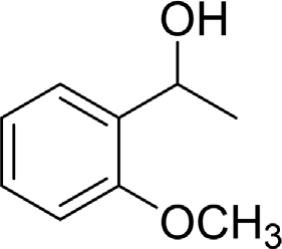	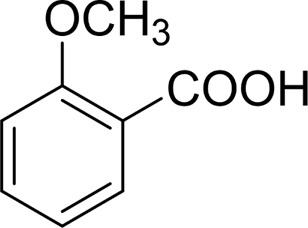 61
9	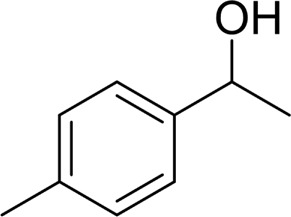	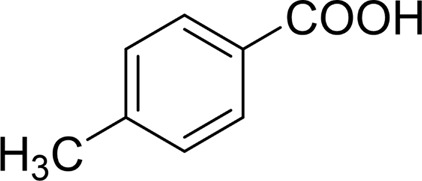 74
10	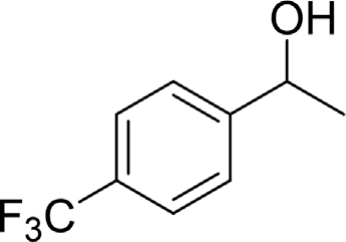	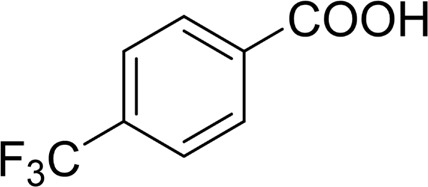 23
11	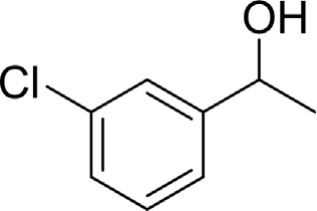	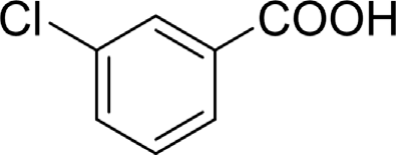 74
12	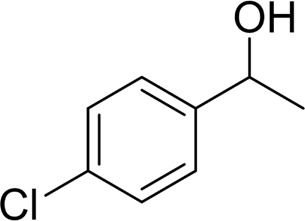	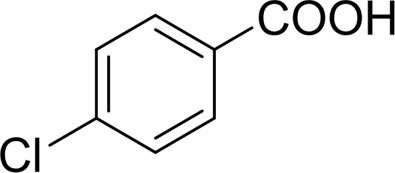 75
13	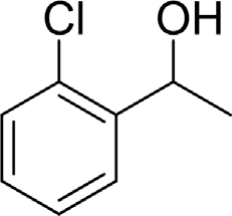	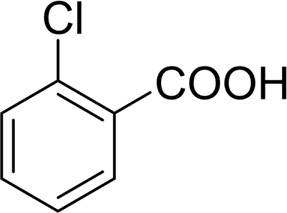 57
14	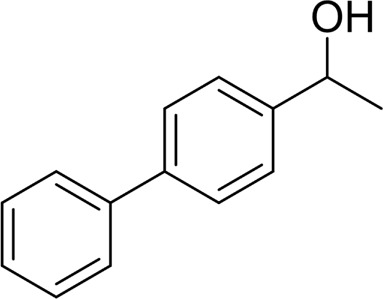	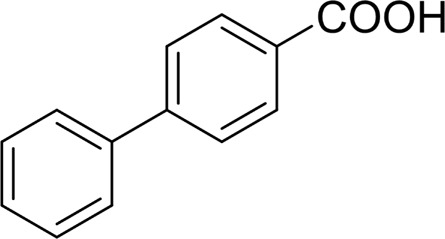 88
15	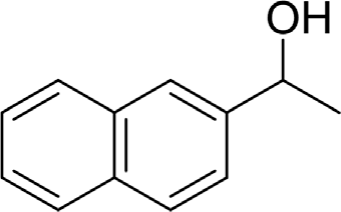	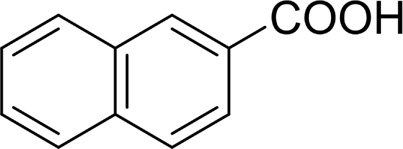 76
16	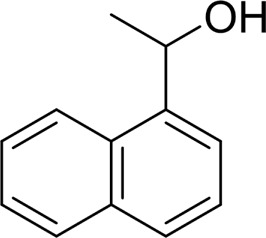	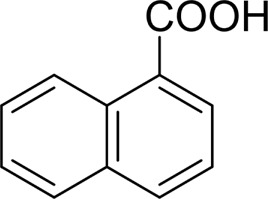 80
17	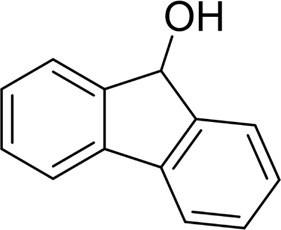	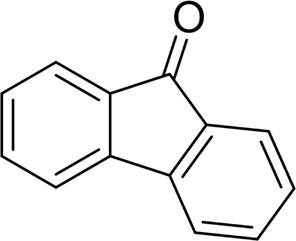 90
18	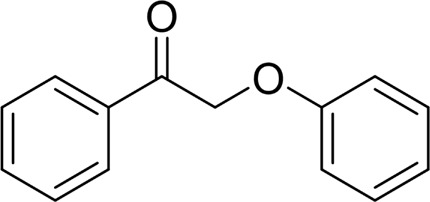	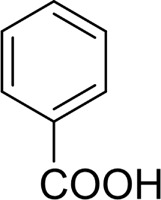 60% 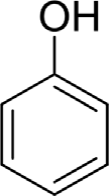 66%

a Condition: substrate (0.5 mmol), Fe(NO_3_)_3_.9H_2_O (0.15 mmol), NaI (0.075 mmol), DMSO (2 ml), and air balloon, 130°C, 18 h.

b Isolated yield.

The aryl alkyl alcohols with different alkyl substituent groups on its β-position of the methyl group were then used as substrates, and all of the aryl alkyl alcohols could be oxidized to desired aryl carboxylic acids in moderate-to-good yields ranging from 42–83%. (Table 2, entries 1–5). Compared with phenyl-1-pentanol, 2-methyl-1-phenyl- propanol with a steric hindrance group on the β-position was transformed into the desired product in 49% yield (Table 2, entry 5). A similar result was obtained from 1-phenyl-2-methyl-1-propanol (42%), which was also with a steric hindrance group in the same position.

In general, substrates with electron-donating or electron-withdrawing groups could be smoothly converted by this catalytic system, and the desired aryl carboxylic acids were obtained in moderate-to-good yields. 1-phenylethanol bearing electron-donating substituents such as 4-CH_3_ and 4-OCH_3_ were transformed to the corresponding benzoic acids in 74 and 74% isolated yields, respectively (Table 2, entries 6 and 9). The 3-OCH_3_ and 2-OCH_3_ substituted 1-phenylethanol gave corresponding benzoic acids in 82 and 61% isolated yields, respectively (Table 2, entries 7–8).

It is obvious that the product from the 2-OCH_3_ substituted substrate is in a lower yield, which might be owing to the ortho-effect. 1-phenylethanol with strong electron-withdrawing groups (4-CF_3_) could also afford the desired product in a low yield (23%) (Table 2, entry 10). In addition, the aryl alkyl alcohols bearing a p- or m-chloro substituent were also transformed into corresponding benzoic acid in moderate yields under standard conditions (Table 2, entries 11 and 12). However, like o-OCH_3_ substituted 1-phenylethanol, the substrate bearing a chloro group in the ortho position was with lower reactivity, and the isolated yield of the product was 57% (Table 2, entry 13).

For 1-phenylethanol derivative with a phenyl substitute group on p-position (Table 2, entry 14), their catalytic oxidation of the substrate to the benzoic acid product resulted in excellent yield (88%). Meanwhile, the 1-(1-naphthyl) ethanol and 1-(2-naphthyl) ethanol also bear an aryl ring substitute with yields upto 80 and 76%, respectively (Table 2, entry 15 and 16). But the 9H-fluoren-9-ol, which was a representative diaryl secondary alcohol, could not be converted to the corresponding acid but to the corresponding ketone in a high yield (93%) (Table 2, entry 17). More interestingly, the important fragment of lignin (Table 2, entry 18) could be converted smoothly to the corresponding benzoic acid and phenol in moderate yield.

Then, various benzyl alcohols and aryl alkyl ketones also could be converted into carboxylic acids in moderate-to-good yields (45–84%) (Table 3, entries 1–8). For example, benzyl alcohols bearing electron-donating substituents such as 4-OCH_3_, 3-OCH_3,_ and naphthyl alcohol substrates were smoothly oxidized to benzoic acid analogs in 69–80% isolated yields. Benzyl alcohols with an electron-withdrawing group (4-CF_3_) could also afford the desired products in a low yield (46%). From the experiments, it was clear that the benzyl alcohols bearing the electron-donating substituent gave higher yields of products than substituted substrates bearing electron-withdrawing substituent, which was similar to the aryl secondary alcohols. Aryl alkyl ketones were also successfully conducted by the Fe(NO_3_)_3_.9H_2_O/NaI/DMSO catalytic system (Table 3, entries 9–11).

**TABLE 3:** Catalytic aerobic oxidation of primary alcohols and ketones^a^.

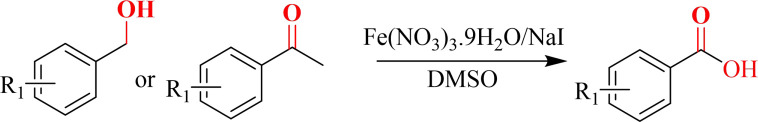		
**Entry**	**Substrate**	**Product/yield** ^b^
1	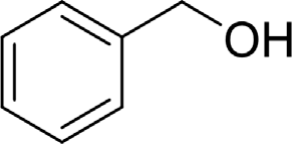	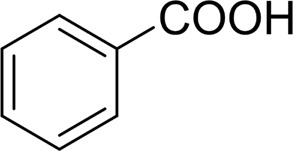 73
2	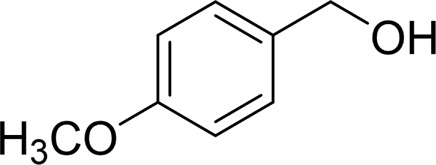	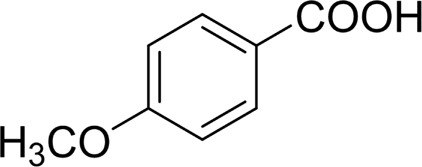 80
3	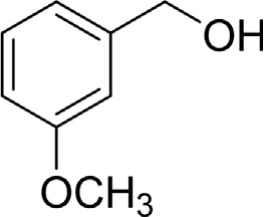	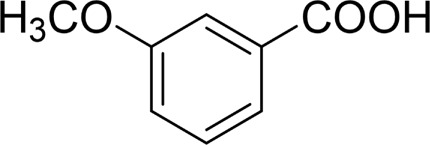 78
4	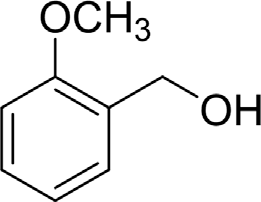	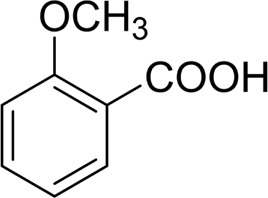 69
5	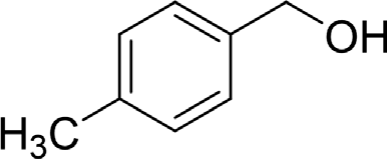	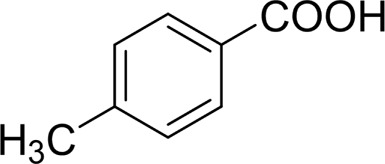 79
6	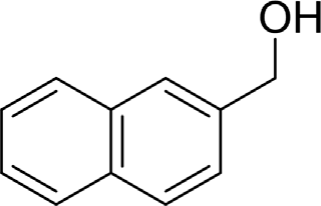	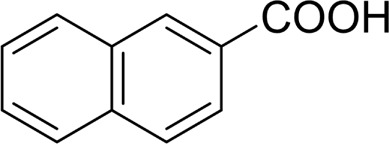 69
7	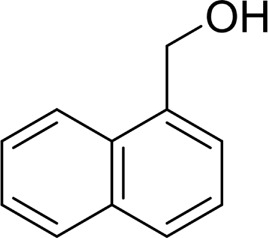	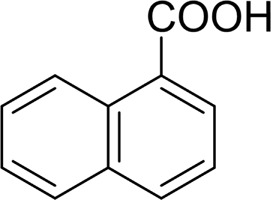 70
8	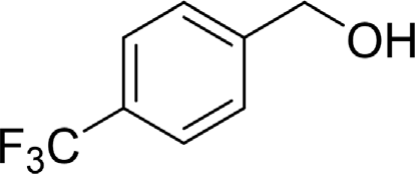	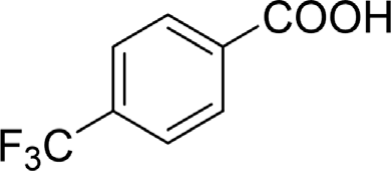 46
9	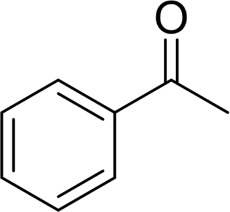	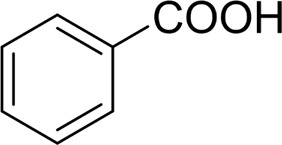 77
10	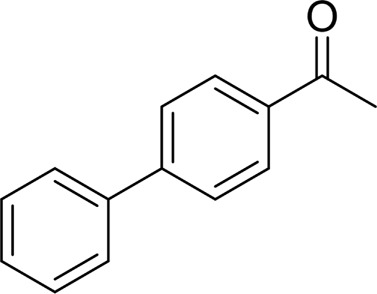	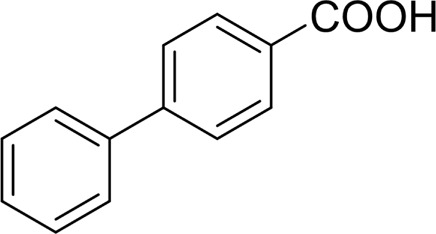 84
11	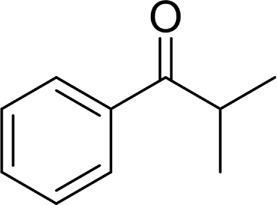	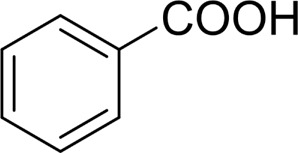

a Condition: substrate (0.5 mmol), Fe(NO_3_)_3_.9H_2_O (0.15 mmol), NaI (0.075 mmol), DMSO (2 ml), and air balloon, 130°C, 18 h.

b Isolated yield.

Next, to gain insight into the mechanism of this oxygenation reaction, some control experiments were set up. Using phenylglyoxal instead of 1-phenyl-1-ethanol as the reactant, it was shown that the phenylglyoxal could be transformed to benzoic acid in 99% yield under standard conditions. Also, benzaldehyde was subjected as the substrate, and a quantitative yield of 99% was produced by benzoic acid under standard conditions. When the reaction was conducted in the absence of NaI, the yield of benzoic acid was low to 56%, while no product was observed without Fe(NO_3_)_3_.9H_2_O. Also, while the reaction was carried out without oxygen, no benzoic acid was obtained, along with a 58% yield of benzaldehyde. From the previous results and the references (Xu et al., 2018a), we proposed a reaction mechanism as follows. Under the reaction condition, the 1-phenylethanol was first oxidized into acetophenone. Initially, I_2_ was generated *in situ* from Fe(NO_3_)_3_.9H_2_O/NaI, and then acetophenone was transformed into a-iodoketone with the help of I_2_ (Elmasry et al., 1998; Naimi-Jamal et al., 2009; Dressen et al., 2009; Martin et al., 2008). Then, a-iodoketone was oxidized into phenylglyoxal by DMSO and releases HI (Wu et al., 2016; Xiang et al., 2016; Wu and Natte, 2016; Xu et al., 2018b). With the Fe salt and H_2_O molecule, the C–C bond was cleaved to give benzaldehyde and release a molecule of formic acid. Benzaldehyde can be oxidized into benzoic acid by the Fe(NO_3_)_3_.9H_2_O/NaI/DMSO catalytic system (see Supplementary Material).

## Conclusion

In summary, the Fe(NO_3_)_3_.9H_2_O/NaI/DMSO catalytic system successfully transformed the aryl alcohols into the desired benzoic acids. In this catalyst system, I_2_ was generated *in situ* from Fe(NO_3_)_3_.9H_2_O/NaI, which was further combined with Fe(NO_3_)_3_.9H_2_O to catalyze the oxidation process. Compared with the Fe(NO_3_)_3_.9H_2_O/I_2_ catalytic system, Fe(NO_3_)_3_.9H_2_O/NaI/DMSO was found with a similar catalytic activity, avoiding direct usage of toxic and corrosive molecular I_2_. Aryl primary and secondary alcohols bearing an electron-donating substituent or electron-withdrawing substituent could be concerted to the corresponding benzoic acid in moderate-to-high yields under standard conditions. Also, the represented lignin model compounds were all successfully transformed into the desired products. Fortunately, it was obvious that the catalytic system had great potential application in terms of green process and technological innovation for the transformation of lignin.

## Data Availability

The original contributions presented in the study are included in the article/Supplementary Material; further inquiries can be directed to the corresponding authors.

## References

[B1] Amin R. Ardeshir K. Heidar Ali A.-N. Zahra T.-R. (2011). Formylation of Alcohol with Formic Acid under Solvent-free and Neutral Conditions Catalyzed by Free I2 or I2 Generated *In Situ* from Fe(NO3)3·9H2O/NaI. Chin. J. Catal. 32, 60–64. 10.1016/s1872-2067(10)60160-x

[B2] Bauer I. Knölker H.-J. (2015). Iron Catalysis in Organic Synthesis. Chem. Rev. 115, 3170–3387. 10.1021/cr500425u 25751710

[B3] Czaplik W. M. Mayer M. Jacobi von Wangelin A. Wangelin J. V. (2010). Iron-Catalyzed Reductive Aryl–Alkenyl Cross-Coupling Reactions. ChemCatChem 3, 135–138. 10.1002/cctc.201000276

[B4] Dressen M. H. C. L. Stumpel J. E. Van de Kruijs B. H. P. Meuldijk J. Vekemans J. A. J. M. Hulshof L. A. (2009). The Mechanism of the Oxidation of Benzyl Alcohol by iron(III)nitrate: Conventional versus Microwave Heating. Green Chem. 11, 60–64. 10.1039/B813030B

[B5] Elmasry M. A. A. Gaber A. Khater E. M. H. (1998). Thermal Decomposition of Ni(II) and Fe(III) Nitrates and Their Mixture. J. Therm. Anal. Calorim. 52, 489–495. 10.1023/a:1010155203247

[B6] Hazra S. Deb M. Elias A. J. (2017). Iodine Catalyzed Oxidation of Alcohols and Aldehydes to Carboxylic Acids in Water: a Metal-free Route to the Synthesis of Furandicarboxylic Acid and Terephthalic Acid. Green Chem. 19, 5548–5552. 10.1039/C7GC02802D

[B7] Iida S. Togo H. (2006). Direct and Facile Oxidative Conversion of Primary, Secondary, and Tertiary Amines to Their Corresponding Nitriles. Synlett 16, 2633–2635. 10.1055/s-2006-951491

[B8] Jiang X. Zhang J. Ma S. (2016). Iron Catalysis for Room-Temperature Aerobic Oxidation of Alcohols to Carboxylic Acids. J. Am. Chem. Soc. 138, 8344–8347. 10.1021/jacs.6b03948 27304226

[B9] Li Y. Xu N. Mei G. Yun Z. Zhao Y. Lyu J. (2018). Fe(NO_3_)_3_·9H_2_O-catalyzed Aerobic Oxidative Deoximation of Ketoximes and Aldoximes under Mild Conditions. Can. J. Chem. 96, 810–814. 10.1139/cjc-2017-0567

[B10] Liu J. Ma S. (2013b). Room Temperature Fe(NO_3_)_3_·9H_2_O/TEMPO/NaCl-catalyzed Aerobic Oxidation of Homopropargylic Alcohols. Tetrahedron 2013 (69), 10161–10167. 10.1016/j.tet.2013.08.082

[B11] Liu J. Ma S. (2013a). Aerobic Oxidation of Indole Carbinols Using Fe(NO3)3·9H2O/TEMPO/NaCl as Catalysts. Org. Biomol. Chem. 11, 4186–4193. 10.1039/C3OB40226F 23677005

[B12] Liu M. Zhang Z. Yan J. Liu S. Liu H. Liu Z. (2020). Aerobic Oxidative Cleavage and Esterification of C(OH)-C Bonds. Chem 6, 3288–3296. 10.1016/j.chempr.2020.09.006

[B13] Martin S. E. Suarez D. (2002). Catalytic Aerobic Oxidation of Alcohols by Fe(NO_3_)_3_–FeBr_3_ . Tetrahedron Lett. 43, 4475–4479. 10.1016/S0040-4039(02)00829-8

[B14] Mohan R. S. Bailey A. D. Cherney S. M. Anzalone P. W. Anderson E. D. Ernat J. J. (2006). A Convenient Method for *In Situ* Generation of I_2_ Using CuSO_4_/NaI and its Applications to the Deprotection of Acetals, Etherifications and Iodolactonizations. Synlett 2, 215–218. 10.1055/s-2005-923586

[B15] Naimi-Jamal M. R. Hamzeali H. Mokhtari J. Boy J. Kaupp G. (2009). Sustainable Synthesis of Aldehydes, Ketones or Acids from Neat Alcohols Using Nitrogen Dioxide Gas, and Related Reactions. ChemSusChem 2, 83–88. 10.1002/cssc.200800193 19115303

[B16] Nicolaou K. C. Chen J. S. Edmonds D. J. Estrada A. A. (2009). Recent Advances in the Chemistry and Biology of Naturally Occurring Antibiotics. Angew. Chem. Int. Ed. 48, 660–719. 10.1002/anie.200801695 PMC2730216 19130444

[B17] Ohlrogge J. Allen D. Berguson B. DellaPenna D. Shachar-Hill Y. Stymne S. (2009). Driving on Biomass. Science 324, 1019–1020. 10.1126/science.1171740 19460990

[B18] Peng J.-B. Qi X. Wu X.-F. (2016). Visible Light-Induced Carbonylation Reactions with Organic Dyes as the Photosensitizers. ChemSusChem 9, 2279–2283. 10.1002/cssc.201600625 27488198

[B19] Perutz M. F. (1979). Regulation of Oxygen Affinity of Hemoglobin: Influence of Structure of the Globin on the Heme Iron. Annu. Rev. Biochem. 48, 327–386. 10.1146/annurev.bi.48.070179.001551 382987

[B20] Plietker B. Dieskau A. (2009). The Reincarnation of the Hieber Anion [Fe(CO) 3 (NO)] - - a New Venue in Nucleophilic Metal Catalysis. Eur. J. Org. Chem. 2009, 775–787. 10.1002/ejoc.200800893

[B21] Rostami A. Rahmati S. Khazaei A. (2009). A Highly Efficient and Ecofriendly Procedure for Tetrahydropyranylation of Alcohols and Phenols in the Presence of *In-Situ* Generated I2 under Heterogeneous and Neutral Conditions. Monatsh Chem. 140, 663–667. 10.1007/s00706-009-0117-7

[B22] Wu X.-F. Natte K. (2016). The Applications of Dimethyl Sulfoxide as Reagent in Organic Synthesis. Adv. Synth. Catal. 358, 336–352. 10.1002/adsc.201501007

[B23] Wu X. Gao Q. Geng X. Zhang J. Wu Y.-d. Wu A.-x. (2016). Iodine-Promoted Oxidative Cross-Coupling of Unprotected Anilines with Methyl Ketones: A Site-Selective Direct C-H Bond Functionalization to C4-Dicarbonylation of Anilines. Org. Lett. 18, 2507–2510. 10.1021/acs.orglett.6b01162 27181791

[B24] Xiang J. C. Cheng Y. Wang M. Wu Y. D. Wu A. (2016). XDirect Construction of 4-Hydroxybenzils via Para-Selective C–C Bond Coupling of Phenols and Aryl Methyl Ketones. Lett. Org. Lett. 18, 4360–4363. 10.1021/acs.orglett.6b02118 27513164

[B25] Xu L. Chen Y. Shen Z. Wang Y. Li M. (2018a). I_2_/Fe(NO_3_)_3_·9H_2_O-catalyzed Oxidative Synthesis of Aryl Carboxylic Acids from Aryl Alkyl Ketones and Secondary Benzylic Alcohols. Lett. Tetrahedron Lett. 59, 4349–4354. 10.1016/j.tetlet.2018.10.060

[B26] Xu L. Wang S. Chen B. Li M. Hu X. Hu B. (2018b). Oxidative C–C Bond Cleavage for the Synthesis of Aryl Carboxylic Acids from Aryl Alkyl Ketones. Synlett 29, 1505–1509. 10.1055/s-0037-1609751

