# Supplementary material for: Catalytic Oxidative Cleavage of C(OH)-C Bonds in Lignin Model Compounds to Carboxylic Acids by Fe(NO3)3.9H2O/NaI/DMSO
Source: Front Chem. 2022 Jul 1;10:933763. doi: 10.3389/fchem.2022.933763 (PMC9283955; doi:10.3389/fchem.2022.933763)
Supplement: Supplementary file 1 [file Table1.DOCX]

Supplementary Material

**I. General experimental details**

**1.1. Materials**

All of the materials were purchased from Beijing Innochem Company, and used as received.

**1.2 Characterization**

Other than the 9H-fluoren-9-ol, mass spectra were obtained on SCIEX X500R QTOF high resolution mass spectromete instrument with negative ion mode. The 0.1 acetic acid/5mM amine acetate in CH_3_CN/H_2_O(20:80) as eluent.

**1.3 General procedures for aerobic oxidation**

Typical procedure: Typical procedure: the desired amount of secondary alcohol substrate (0.5 mmol), Fe(NO_3_)_3_**^.^**9H_2_O (0.15 mmol), NaI (0.075 mmol), DMSO (2 mL) were added into a 25 mL reaction bottle. Then the mixture was degassed though three times with the oxygen balloon, the reaction was hold under 130 ^o^C for the desired time. After being acidificated with 2 mol/L HCl (3 mL), the solution was extracted by ethyl acetate (5 mL) twice, the organic phase was washed with saturated brine once and dried by Na_2_SO_4_. The combined organic phase was removed the solvent by a rotary evaporator. The desired product was obtained through column chromatography using ethyl acetate/petroleum ether as eluent.

**II. HPLC-MS and NMR**

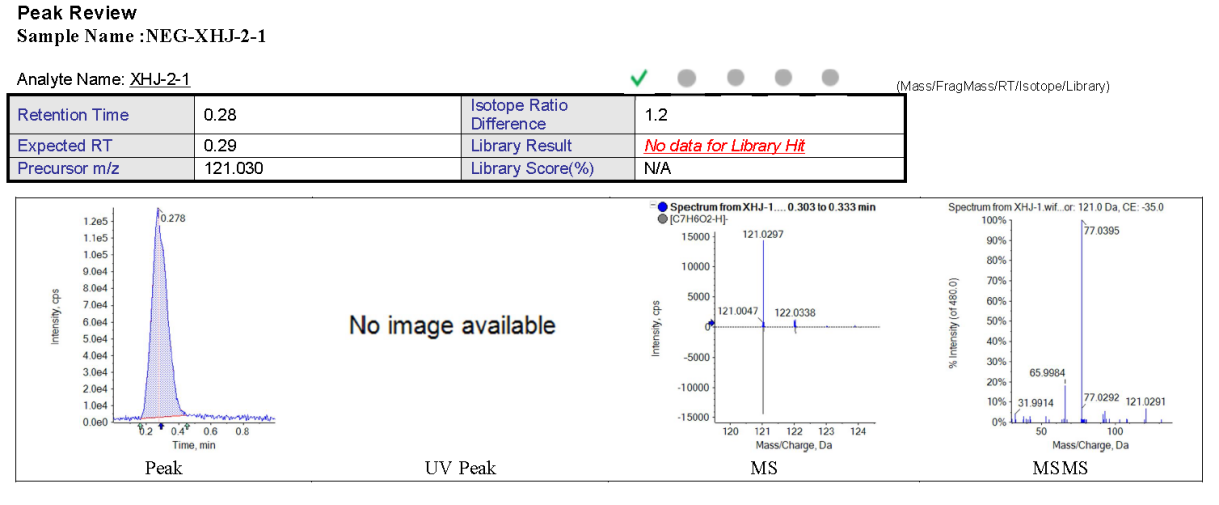


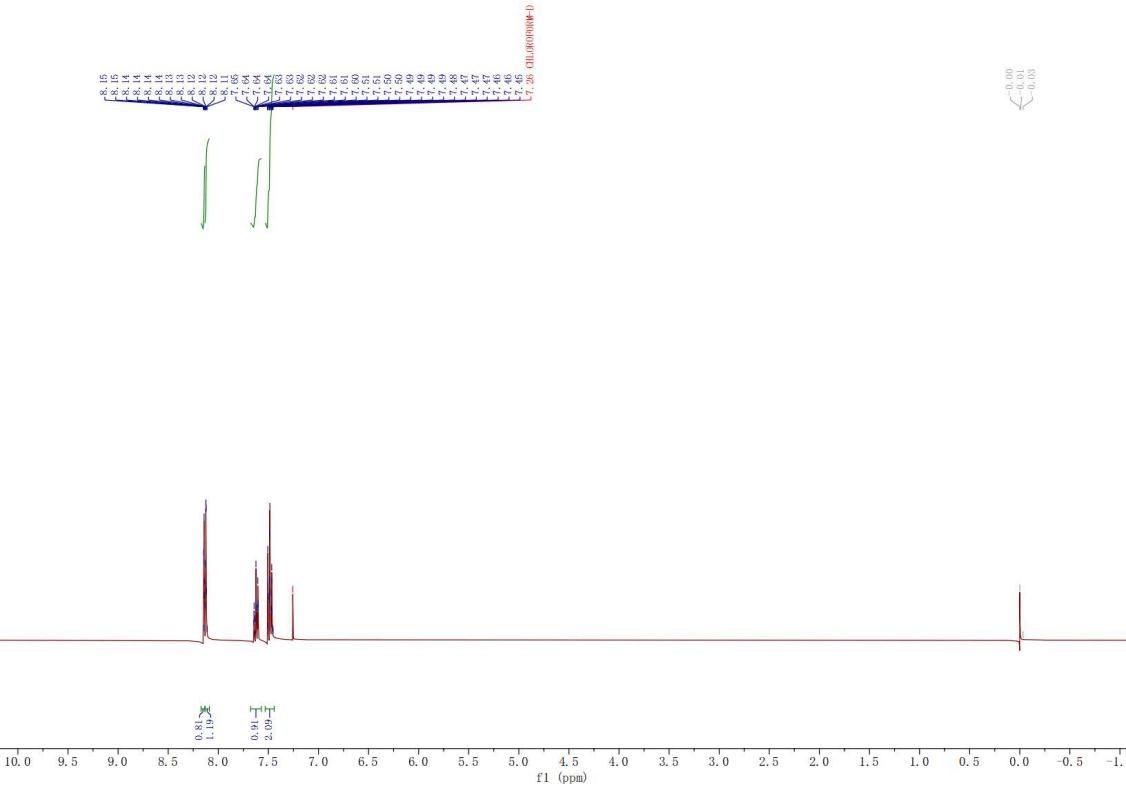


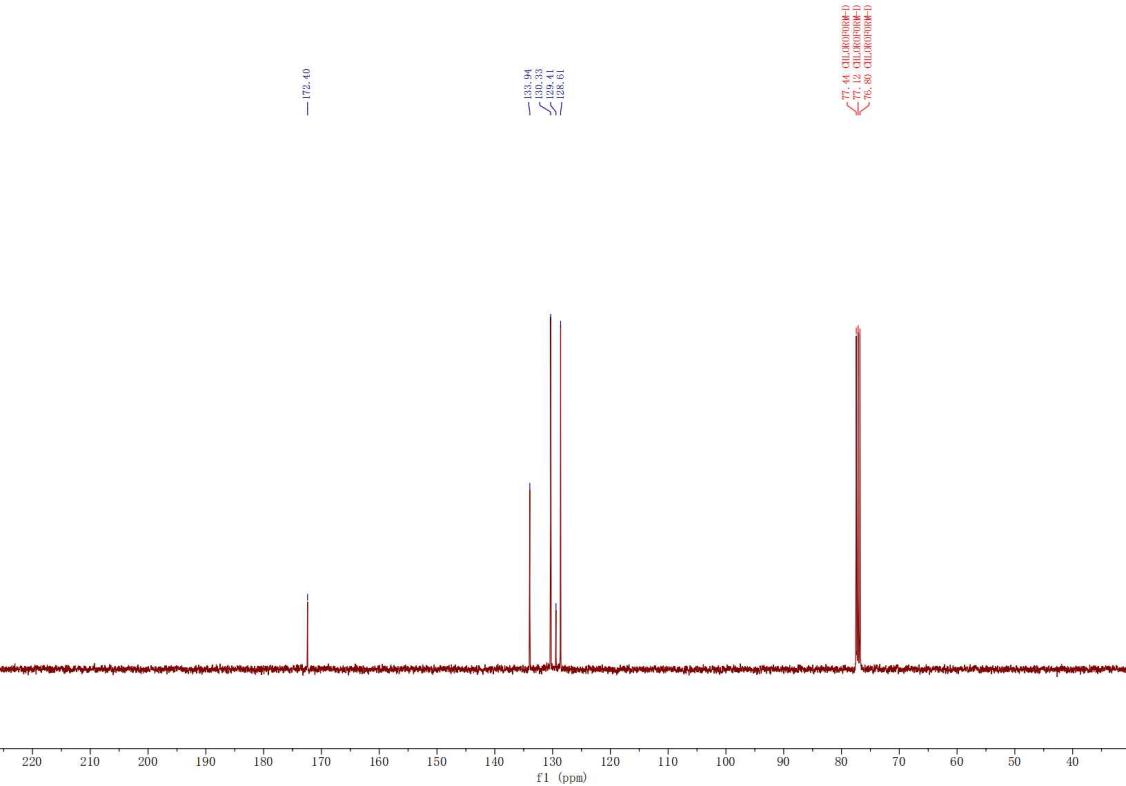

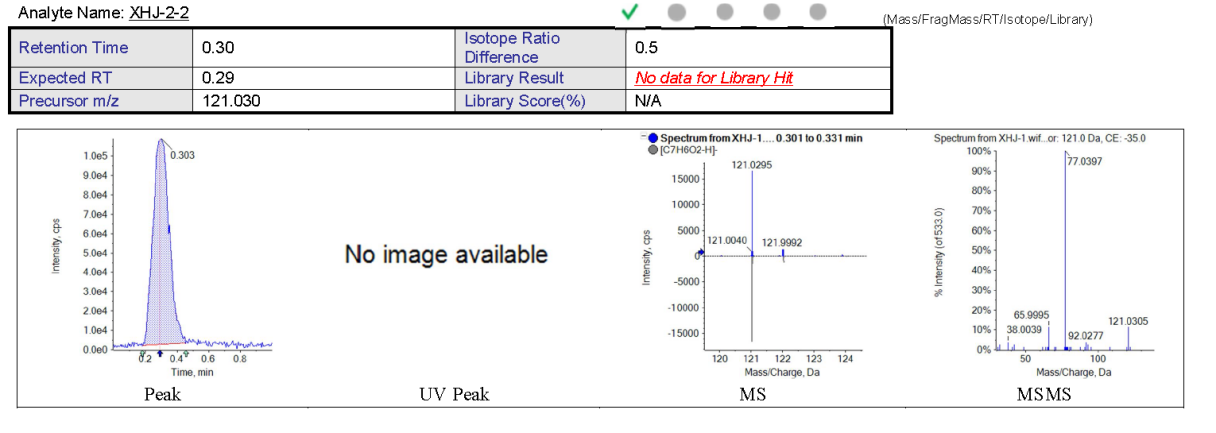

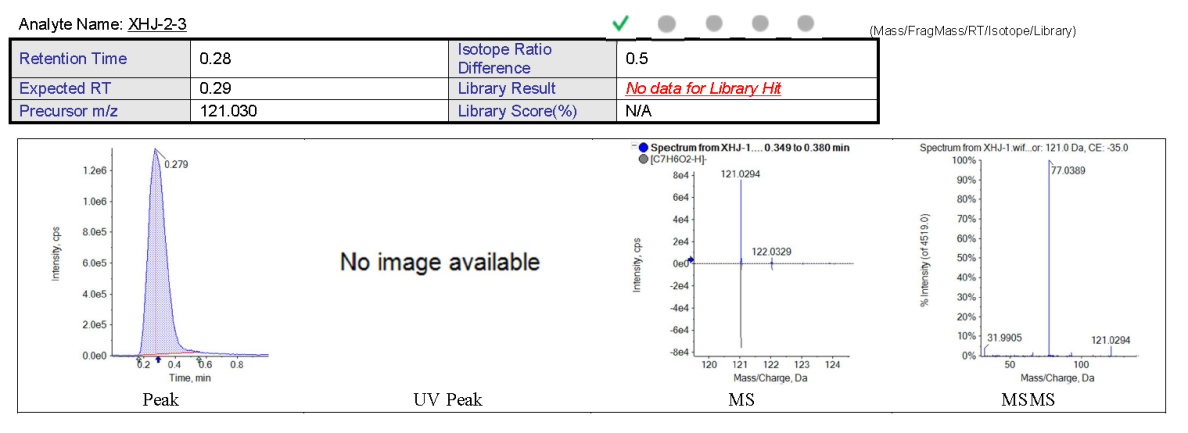

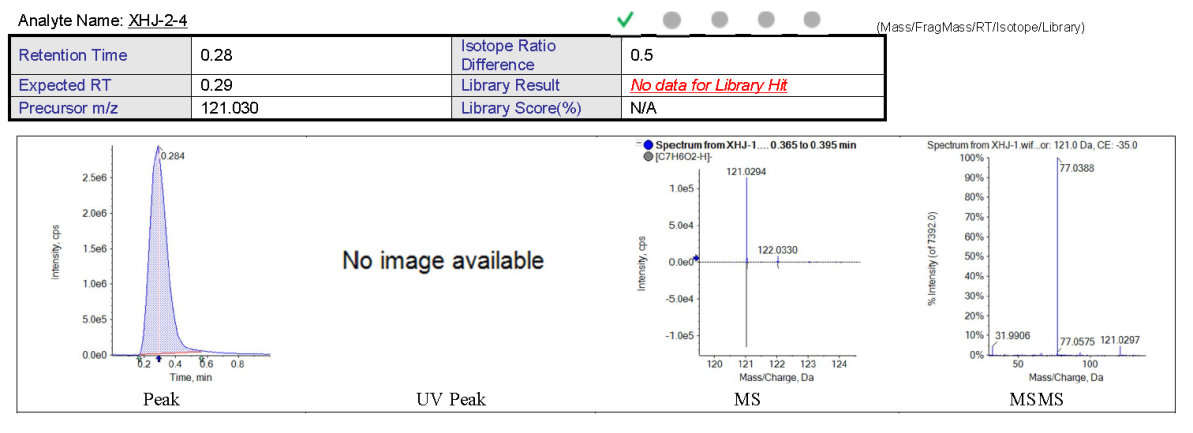

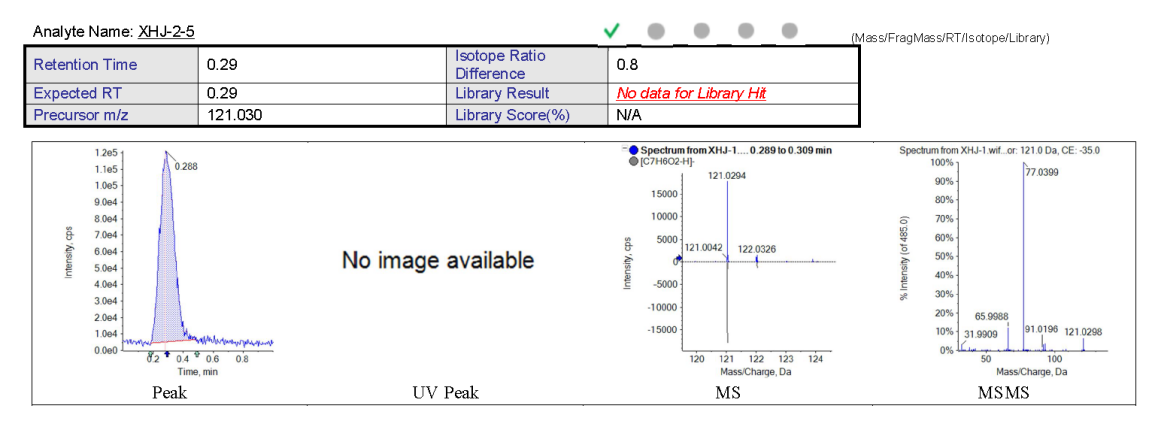

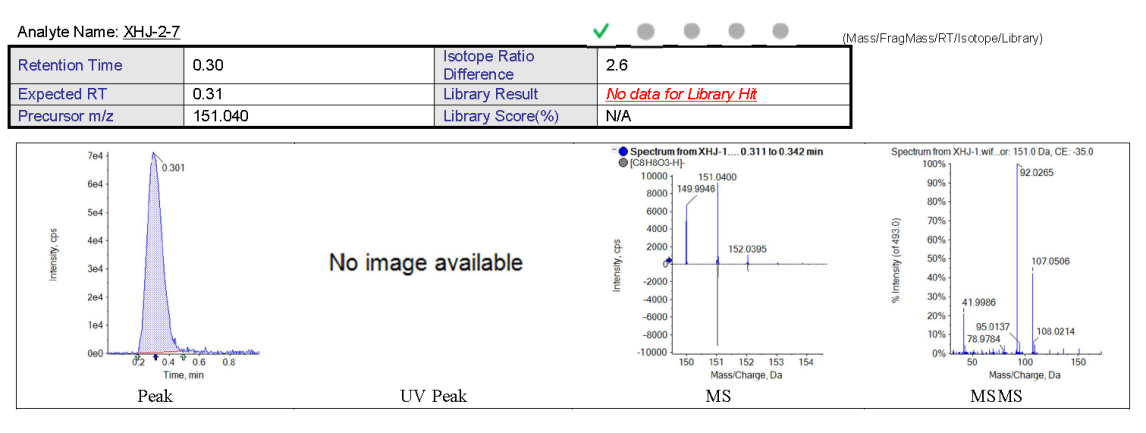


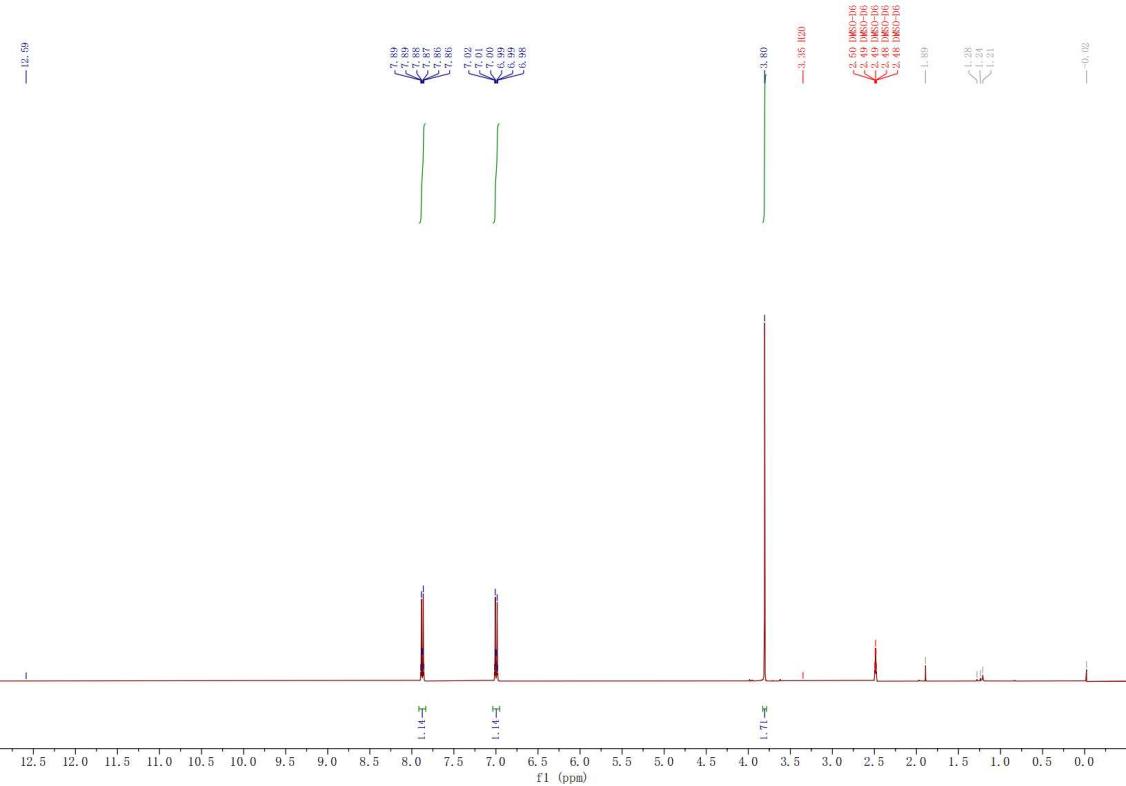


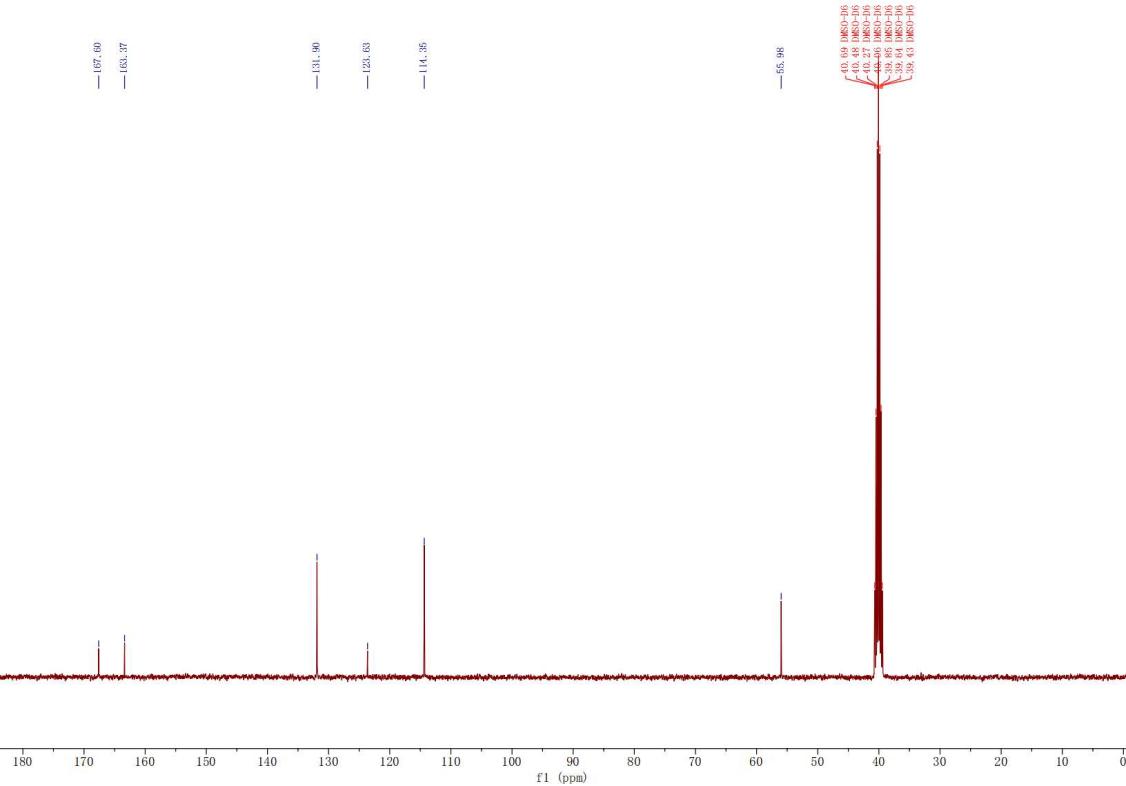

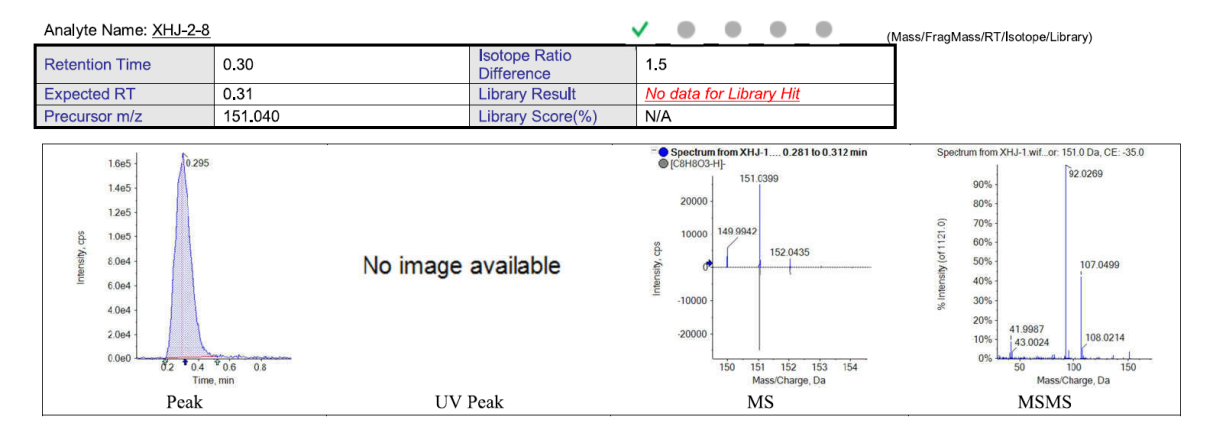

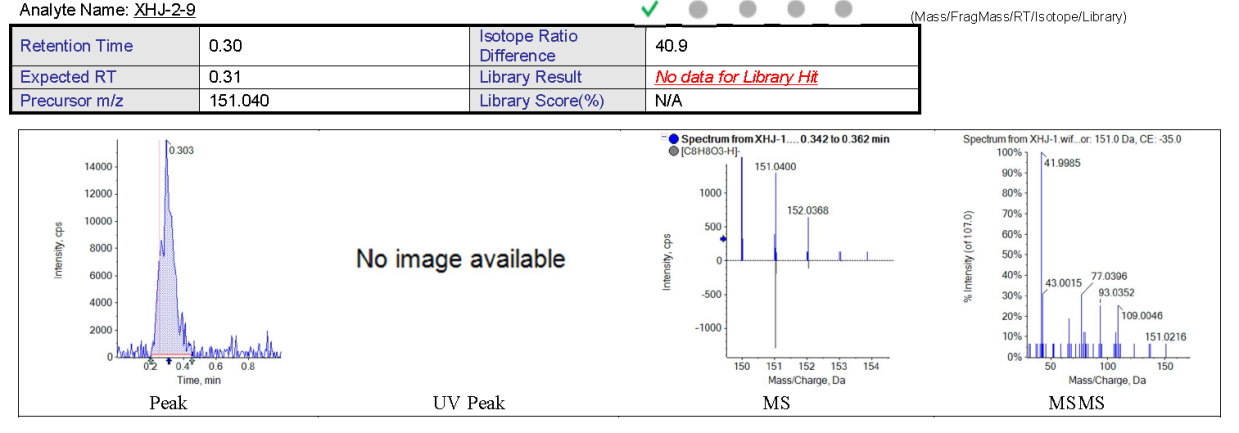

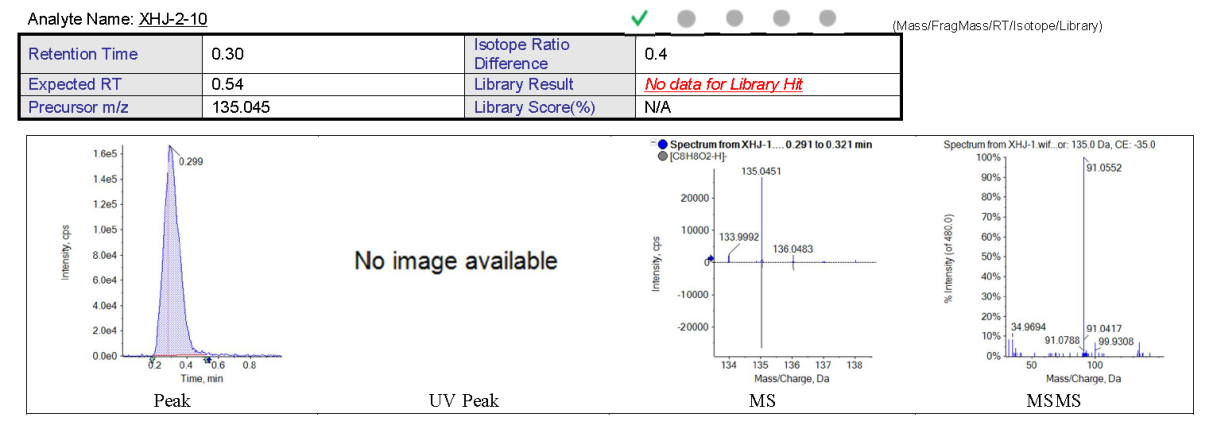

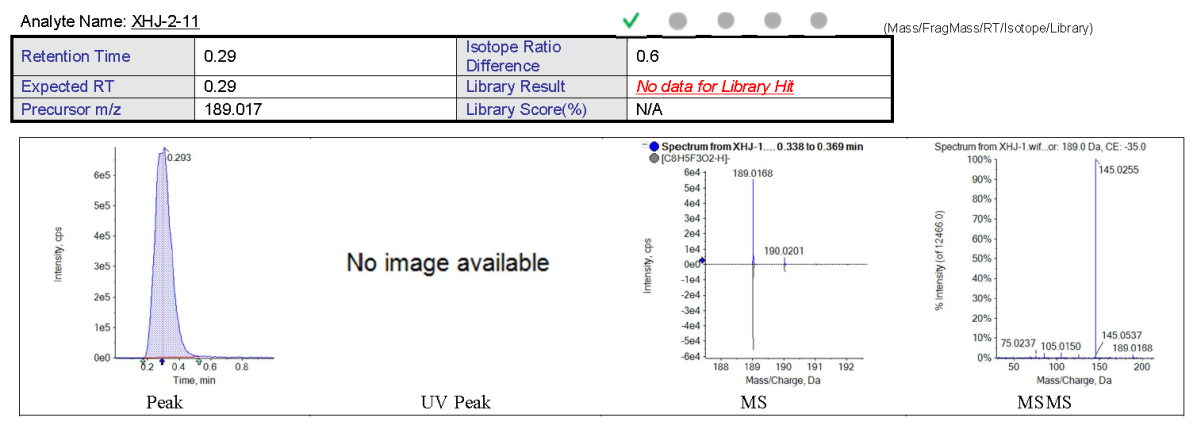

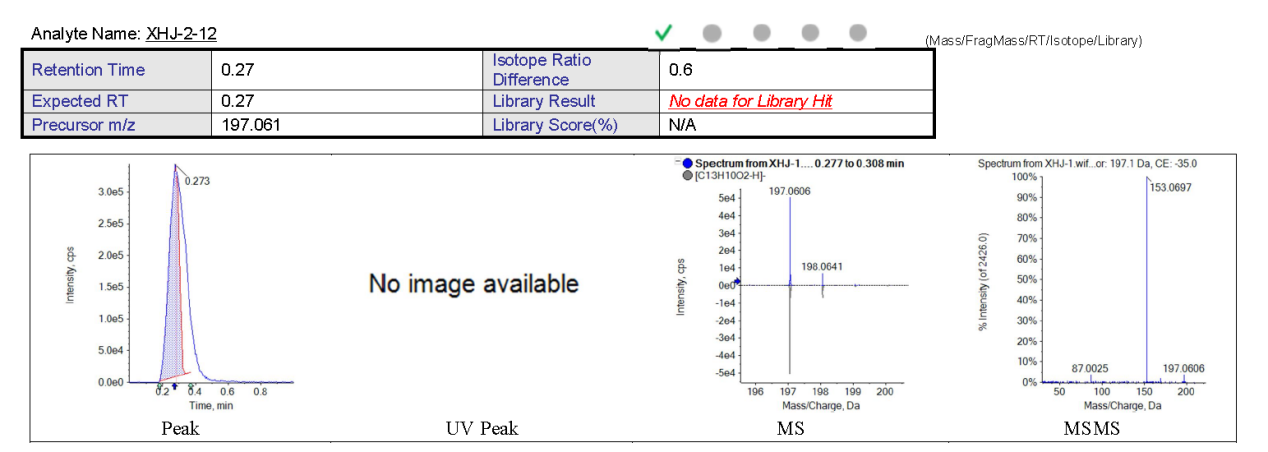

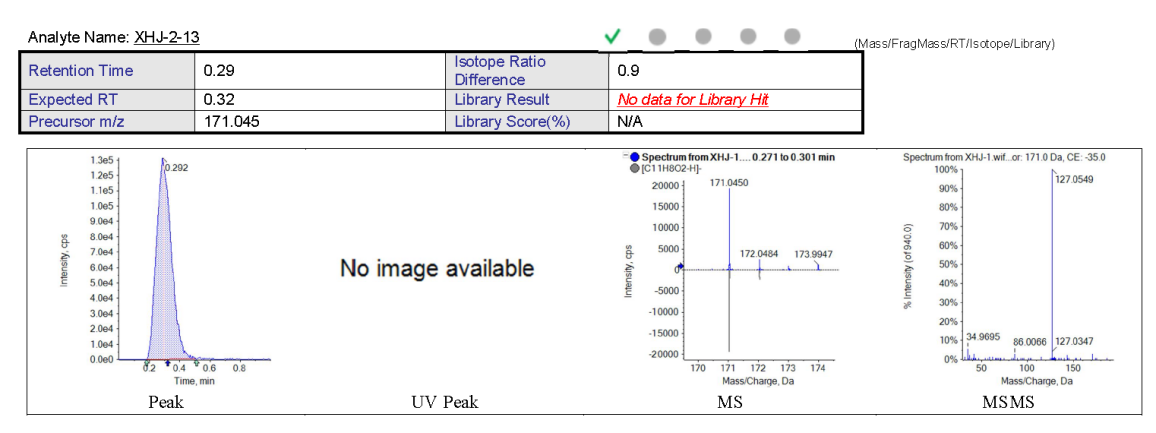

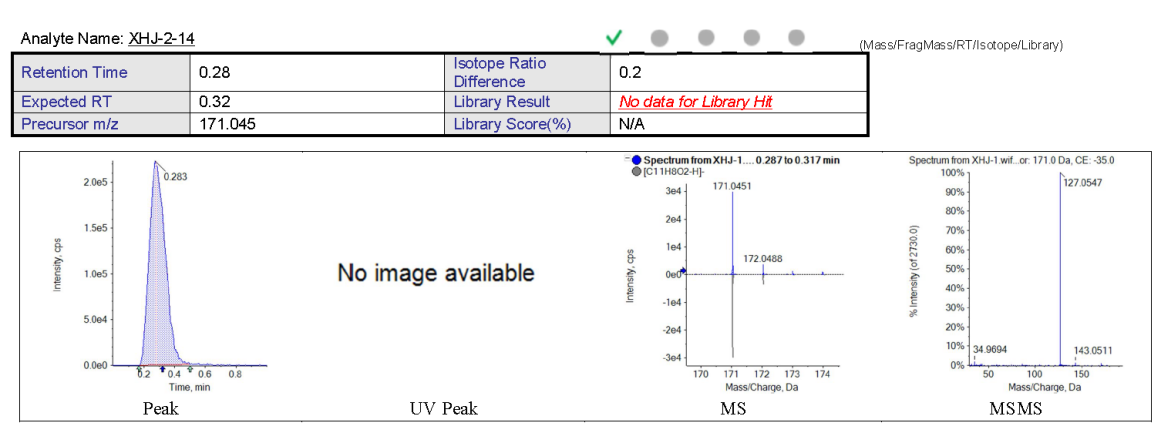

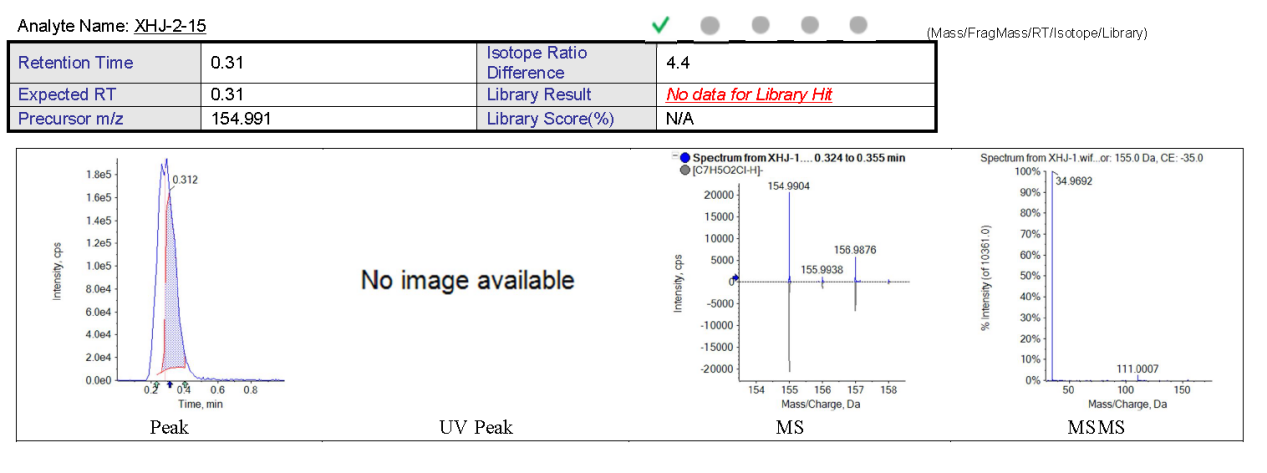


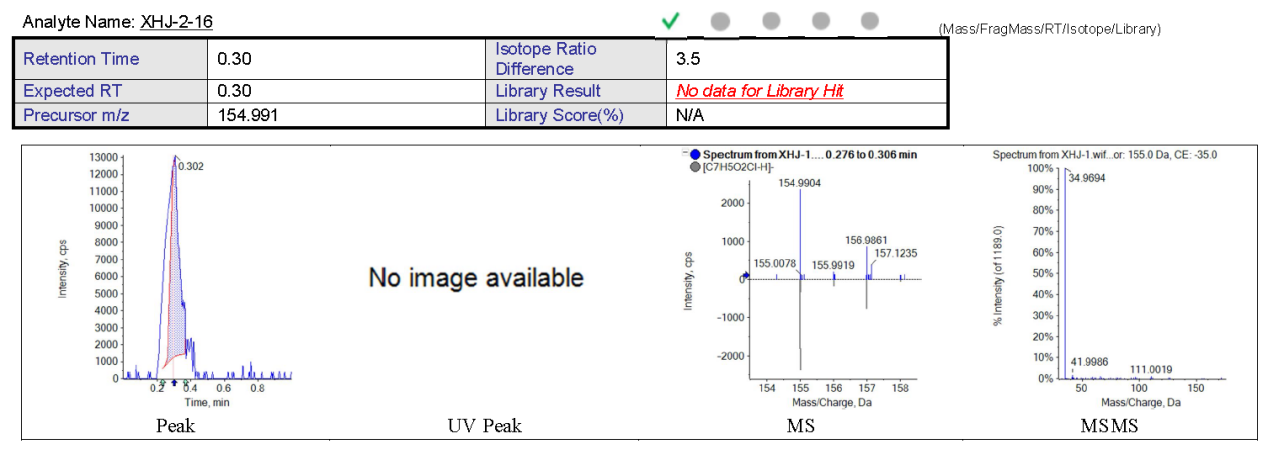

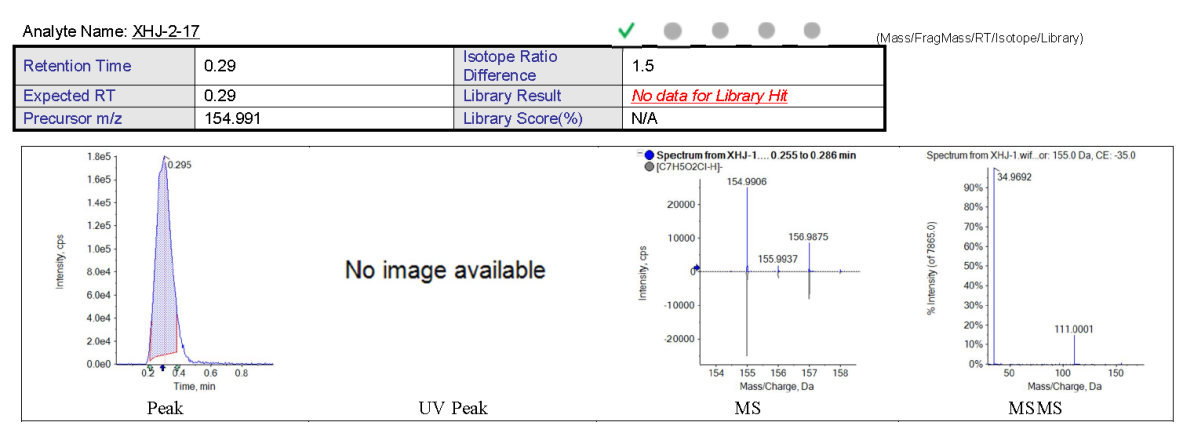

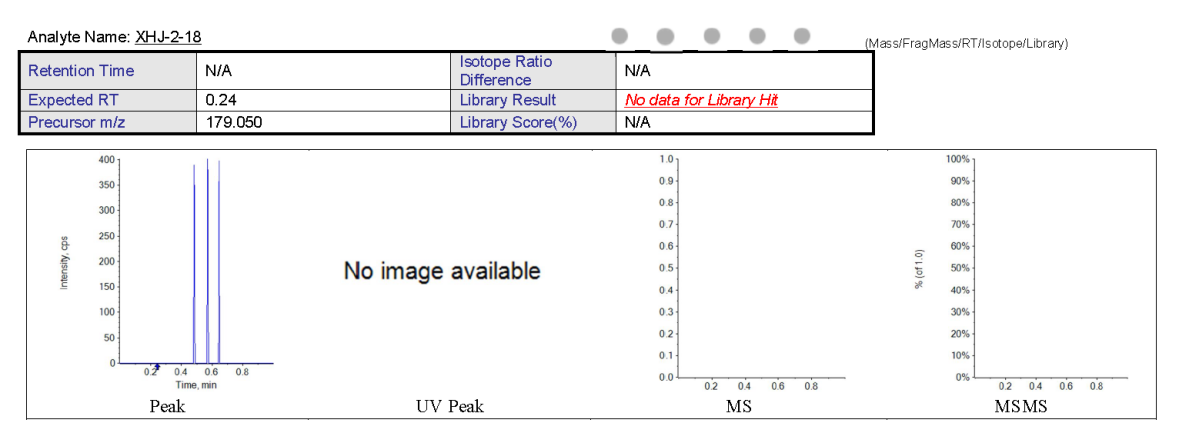

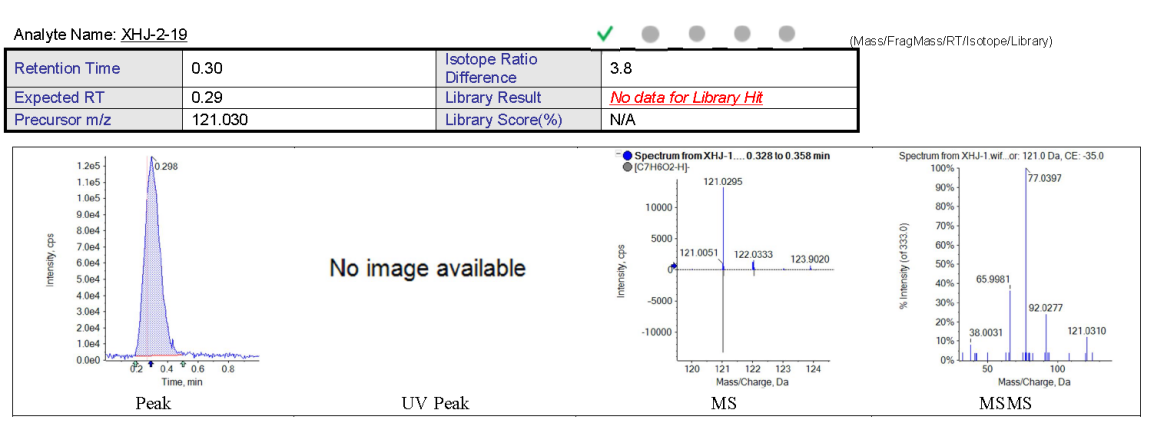

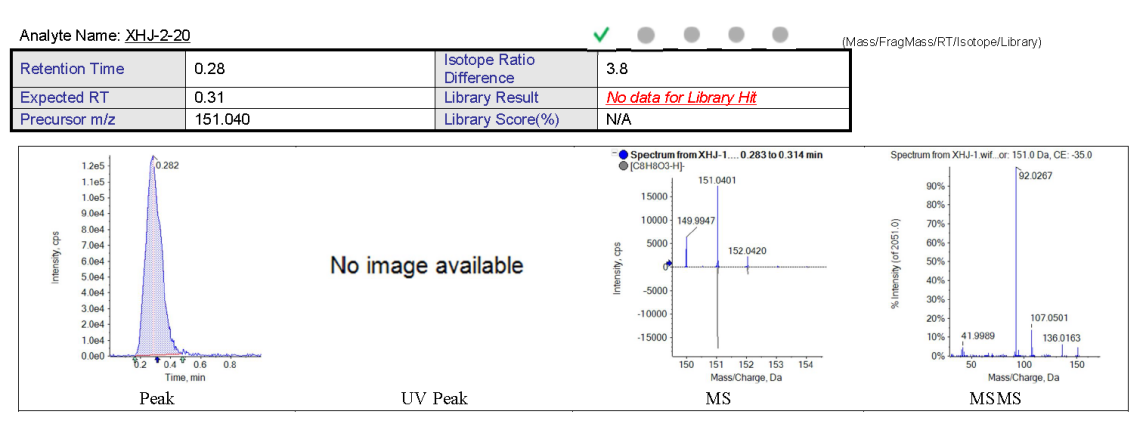

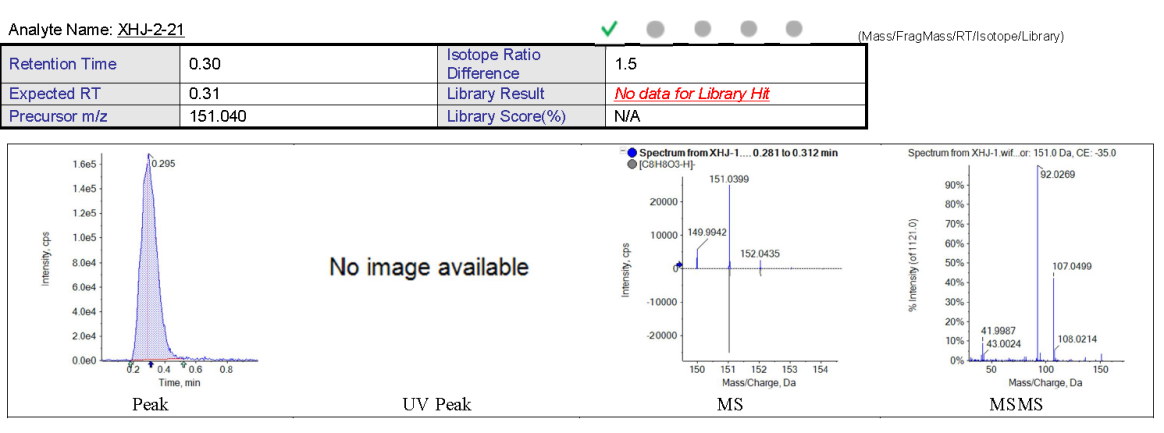

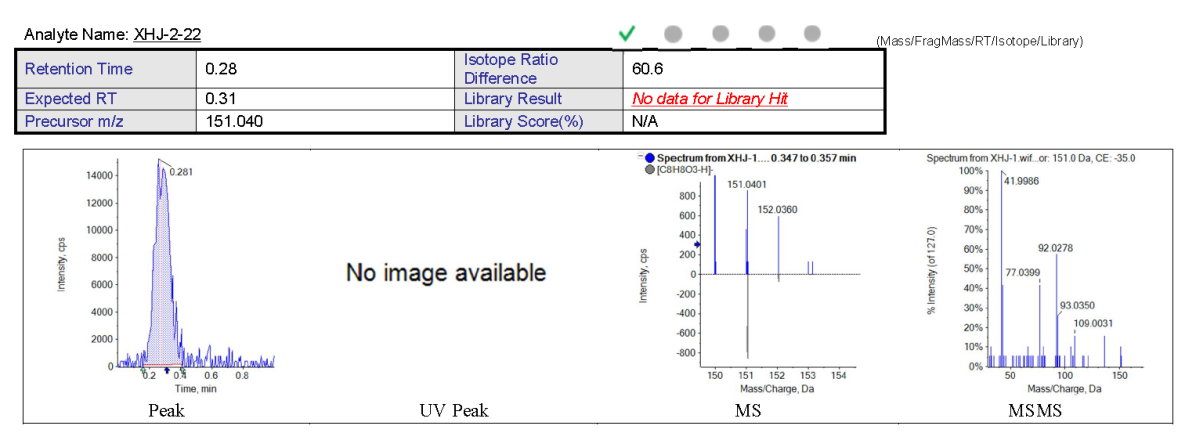

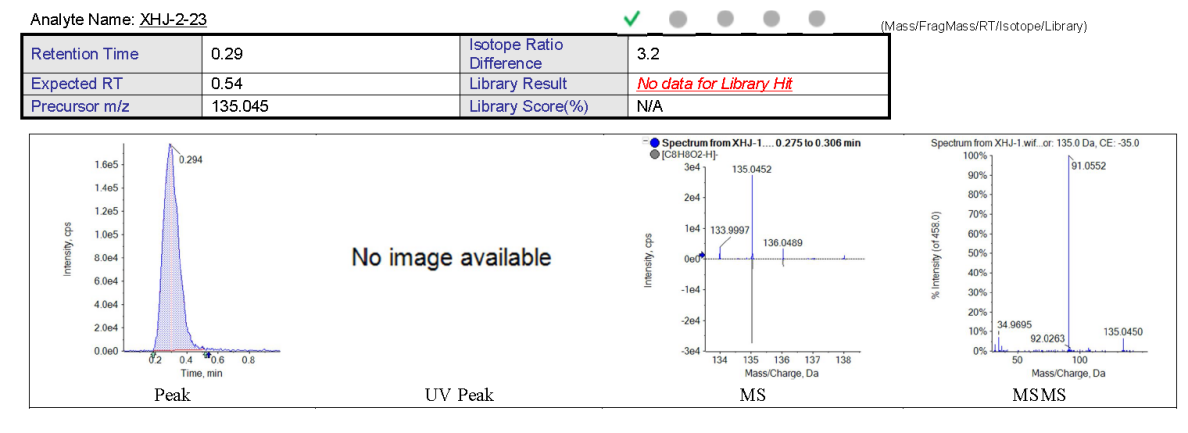

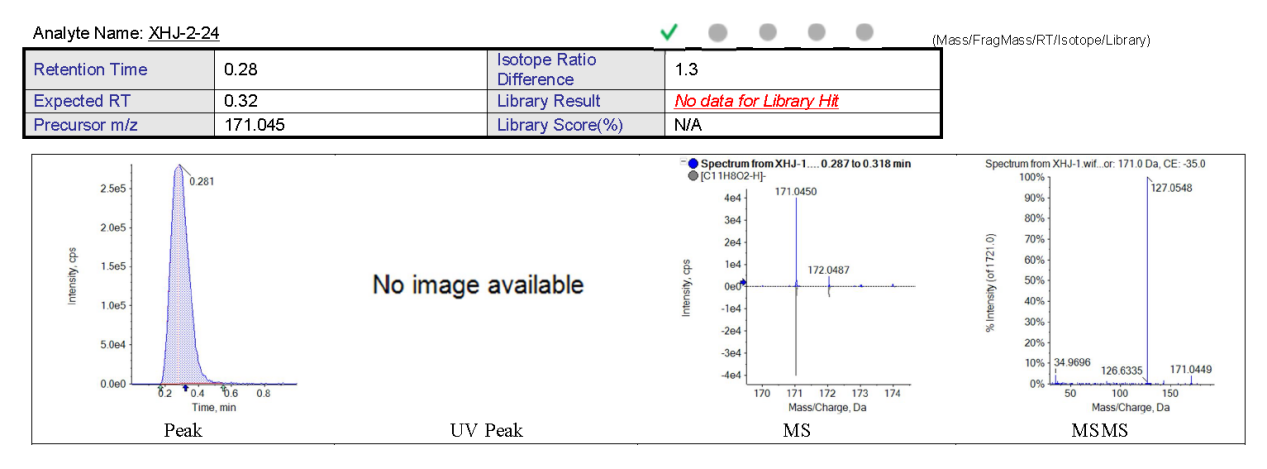

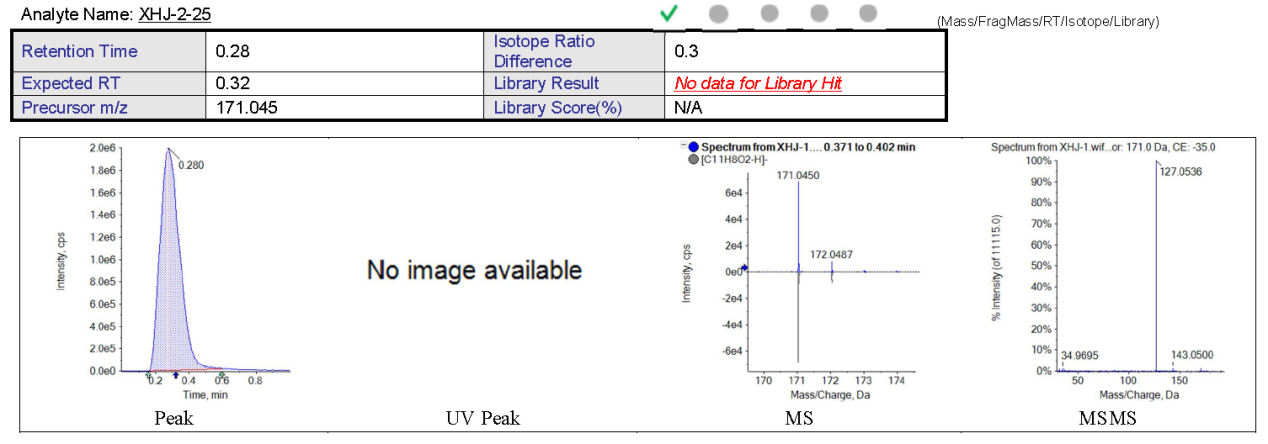

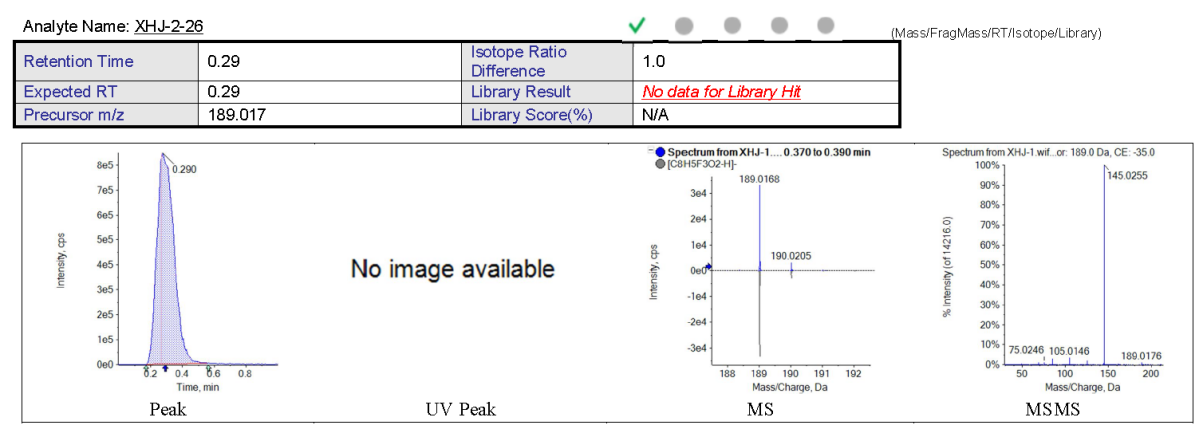

from Fe(NO_3_)_3_^.^9H_2_O/NaCl/DMSO


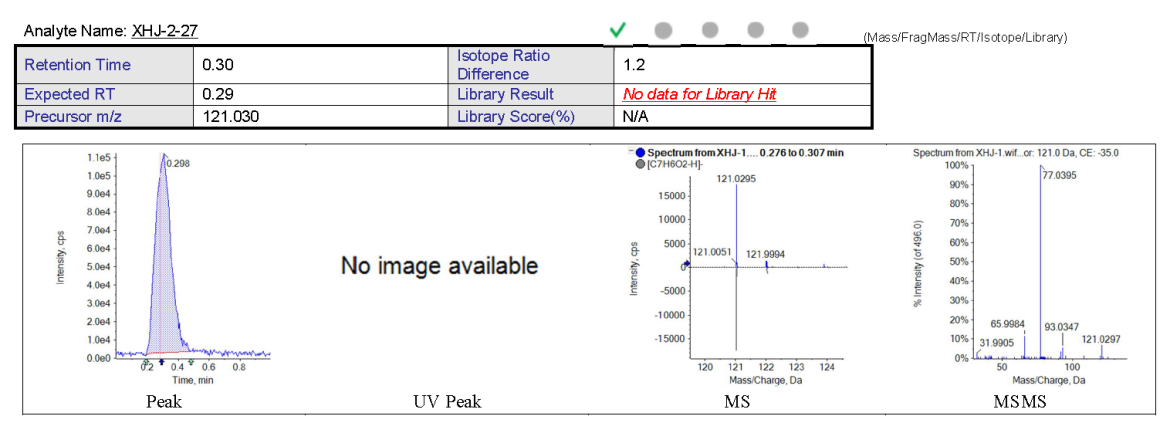

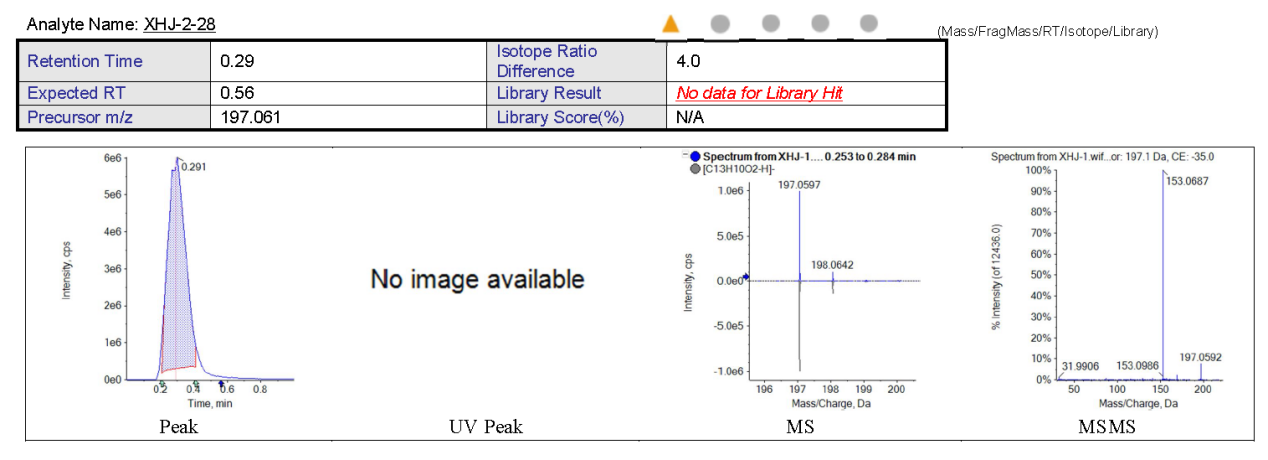

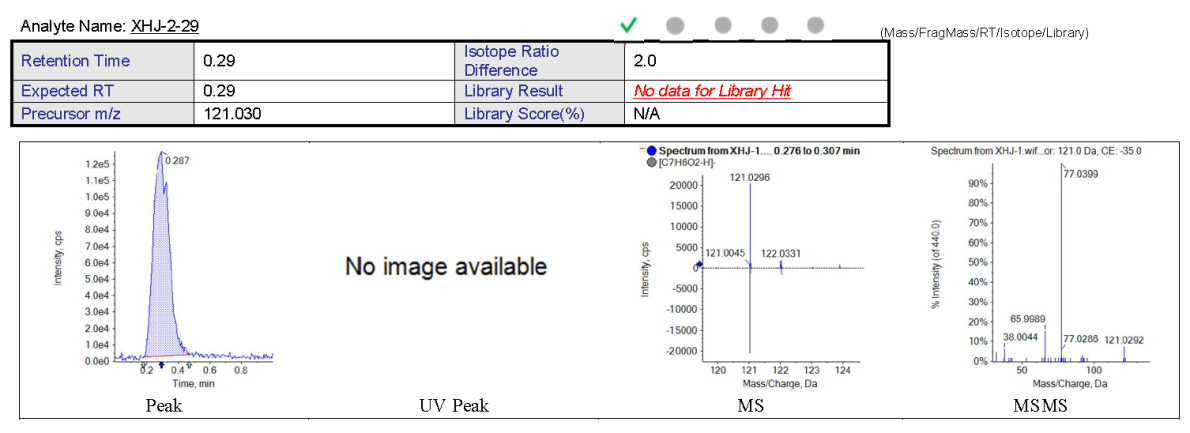

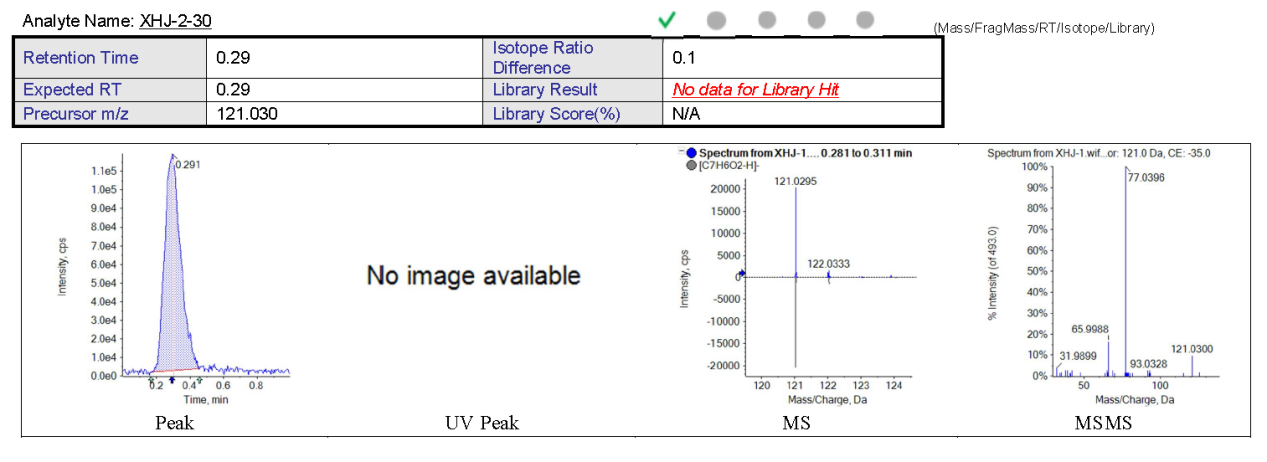

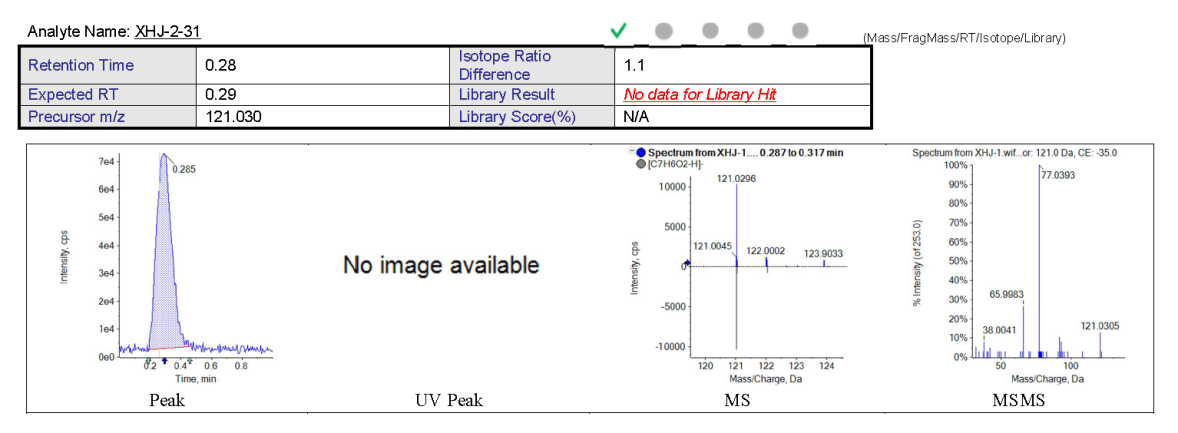

Scheme 1. Plausible reaction pathways
